# The myriapodological legacy of Victor Ivanovich Motschoulsky (1810–1871)

**DOI:** 10.3897/zookeys.426.8011

**Published:** 2014-07-17

**Authors:** Sergei Golovatch

**Affiliations:** 1 Russian Academy of Sciences, Institute for Problems of Ecology and Evolution, Leninsky pr. 33, 119071 Moscow, Russia

**Keywords:** Myriapoda, taxonomy, Zoological Museum, Moscow, Russia

## Abstract

The little that remains of Motschoulsky’s myriapodological legacy in the collection of Moscow’s Zoological Museum proves to be of very limited value. Only one species of Diplopoda described by Motschoulsky, the Caucasian *Hirudisoma roseum* (Victor, 1839), is still in use, yet requiring a neotype designation, whereas the remaining few myriapod names he proposed are either *nomina dubia* or *nomina nuda*. The former include *Scolopendra pentagramma* Motschoulsky, 1866 (Chilopoda, Scolopendromorpha, Scolopendridae) and *Strongylosoma carinulatum* Motschoulsky, 1866 (Diplopoda, Polydesmida, Paradoxosomatidae), both from Japan, as well as *Julus costulatus* Motschoulsky, 1851 (Diplopoda, Callipodida, Schizopetalidae?), from Montenegro, because their type material is either inadequate or missing.

## Introduction

**Figure 1.**
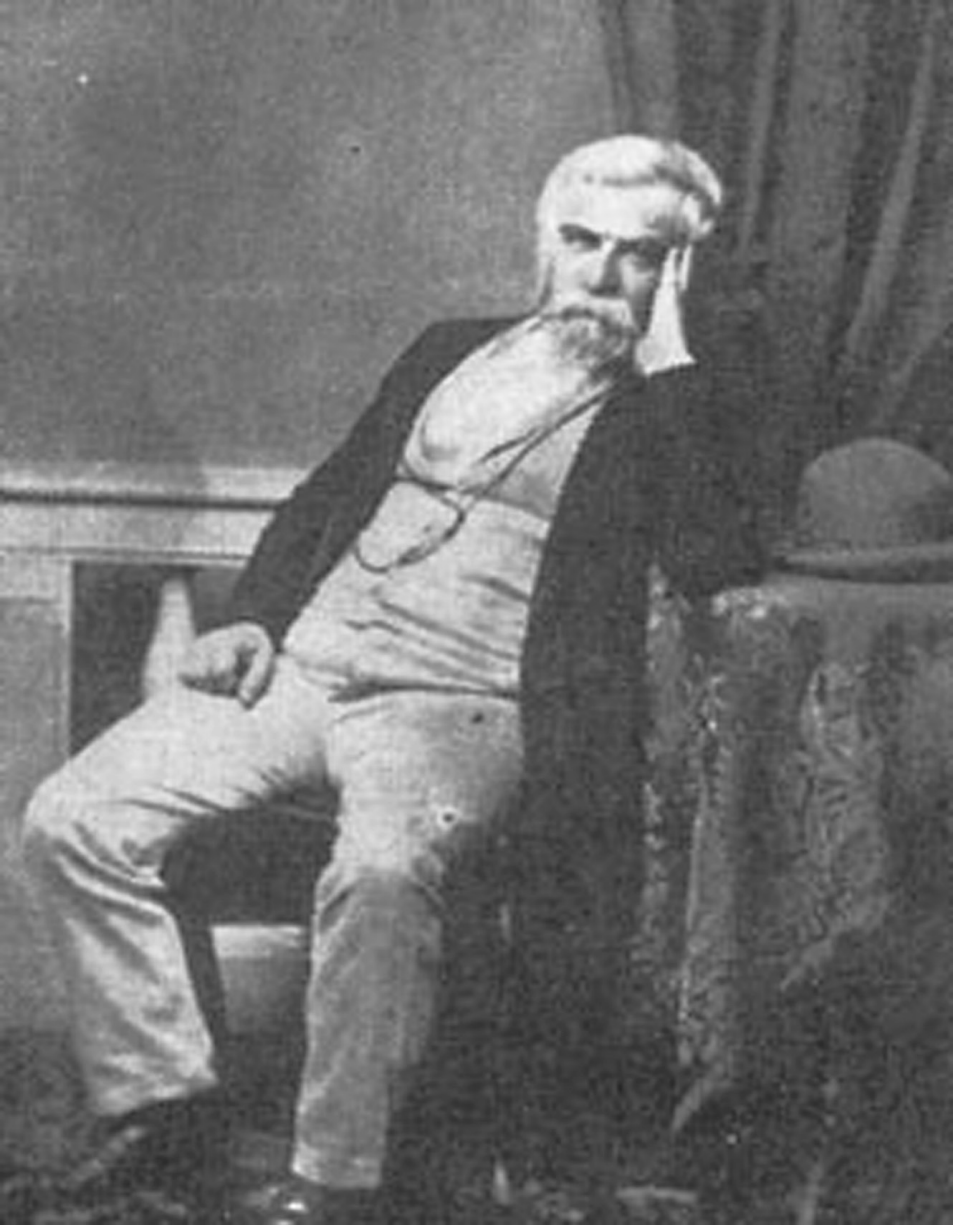
Portrait of Victor Ivanovich Motschoulsky.

With the recent publication of V. I. Motschoulsky’s memoirs (Krivokhatsky 2013) and several reviews of that book (Mikhailov 2013, 2014, Mikhailov and Golovatch 2014), public interest to his legacy has considerably revived. Motschoulsky (= Motschulsky) is best known as a prominent Russian entomologist of his time, mostly a specialist in beetles (Fig. 1). His collection is currently kept in the Zoological Museum of the State University of Moscow, Russia (ZMUM).

Motschoulsky’s published contributions to myriapodology are very few, but even these have largely remained neglected. To my knowledge, no-one has ever attempted to revise any of Motschoulsky’s myriapod types.

## Material

**Figure 2.**
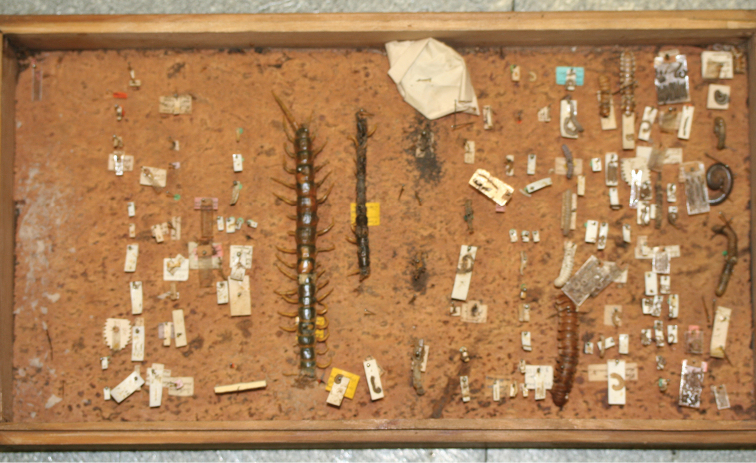
Picture showing Motschoulsky’s box with pinned dry Myriapoda (ZMUM collection).

The Moscow Museum collection of Myriapoda still contains Motschoulsky’s original wooden box full of dry pinned animals (Fig. 2)! There are a few dozen species of Diplopoda, many of which exotic (e.g. Andrognathidae, Platydesmida or Platyrhacidae, Polydesmida), and only a couple of larger Scolopendridae specimens. Much of the material is in poor condition, damaged by dermestid beetle larvae.

## Results

Despite such a profound diversity, most of the diplopods are devoid of labels. Those which are labeled are either identified (e.g. “*Polydesmus complanatus* (L.)” or “*Scolopendra spinula* Brandt”) or bear provisional names marked as “mihi”. These latter specimens must be considered as *nomina nuda*. One such name has even been published: *Julus melanocephalus* Motschoulsky, 1851, said to be very common across Montenegro, even though specimens were clearly smaller near Cattaro (Motschoulsky 1851: 595). Since neither a diagnosis nor a description has been provided, that *nomen nudum* has rightly been ignored ever since.

The few samples of “*Polyzonium germanicum* Brandt” contained in the ZMUM box may well represent the material Motschoulsky (1853) reported from Valaam Island, White Sea, Russia’s North (Russian translation: Krivokhatsky 2013: 176). Their identification is correct.

Motschoulsky, sometimes under the pseudonym “Victor”, is known to have published only four valid myriapod species. The earliest is *Leiosoma rosea* (recte: -*um*) Victor, 1839, from eastern Georgia, Caucasus (Victor 1839), properly redescribed from additional samples from Transcaucasia much later (Lignau 1911). Since the name *Leiosoma* Victor, 1839, established for *Leiosoma roseum* by monotypy, is preoccupied by *Leiosoma* Stephens, 1829 (Coleoptera) (Jeekel 1970), most of the subsequent references to *Leiosoma roseum* (e.g. Lohmander 1936, Kobakhidze 1965) are incorrect. The species currently belongs to *Hirudisoma* Fanzago, 1881 (e.g. Latzel 1884 et auctorum) and is now referred to as *Hirudisoma roseum* (Victor, 1839) (e.g. Lokšina and Golovatch 1979). Even though no type material of that species could be traced in the ZMUM box, the identity of *Hirudisoma roseum*, a presumed Caucasian endemic, will become unquestioned as soon as a neotype is designated in the future.

The second valid diplopod name proposed by Motschoulsky (1851), *Julus costulatus* Motschoulsky, 1851, from the environs of Cattaro (= Kotor), Montenegro, has long been considered as a putative synonym of either *Apfelbeckia insculpta* (L. Koch, 1867) or *Acanthopetalum carinatum* (Brandt, 1840) (Latzel 1884). Since no type material of *Julus costulatus* has been revealed in the ZMUM box, the species is bound to remain a *nomen dubium* (Stoev et al. 2008).

A careful search for type material has revealed two type series only: the holotype of *Scolopendra pentagramma* Motschoulsky, 1866 (ZMUM ρ7449) and 3 syntypes of *Strongylosoma carinulata* (recte: -*tum*) Motschoulsky, 1866 (ZMUM ρ2376), both species coming from unknown localities in Japan. As their original descriptions (Motschoulsky 1866) are anecdotal to realistically make these species recognizable (Pocock 1895, Kraepelin 1903), they have been completely neglected/omitted ever since (e.g. Attems 1914, 1937, Jeekel 1968, Nguyen and Sierwald 2013). Unfortunately, the existing types of both *Scolopendra pentagramma* and *Strongylosoma carinulatum* appear to be of too little value to reveal the identities of these species. Thus, both ends of the *Scolopendra pentagramma* holotype have been so badly destroyed that all that can be said is that it may well be a *Scolopendra* Linnaeus, 1758, but it is certainly not a *Cormocephalus* Newport, 1844, because it has tarsal spurs (J. Lewis, personal communication). NB: Only three species of *Scolopendra* are currently known to occur in Japan, *Scolopendra morsitans* Linnaeus, 1758, *Scolopendra subspinipes* (Leach, 1815) and *Scolopendra multidens* Newport, 1844 (P. Stoev, personal communication). In this case, Motschoulsky’s *Scolopendra pentagramma*, if identifiable, would anyway represent a junior synonym of one of the above three species.

Similarly, all 3 syntypes of *Strongylosoma carinulatum* (one, lacking a few caudalmost segments, is still pinned, the remaining two have been located among the debris at the bottom of the ZMUM box) are later juvenile instars of a paradoxosomatid millipede, thus being absolutely inapt for revealing the species’ identity. In other words, both *Scolopendra pentagramma* Motschoulsky, 1866 and *Strongylosoma carinulatum* Motschoulsky, 1866 are also doomed to remain *nomina dubia*.

## Conclusion

To summarize, the little that remains of Motschoulsky’s myriapodological legacy in the collection of the Moscow Museum proves to be of very limited value. However, even the negative result is a result. Only one species of Diplopoda described by Motschoulsky, the Caucasian *Hirudisoma roseum* (Victor, 1839), is still in use, yet requiring a neotype designation, whereas the remaining few myriapod names he proposed are either *nomina dubia* or *nomina nuda*.
